# Skeletal muscle alpha actin acetylation enhances myosin binding and increases calcium sensitivity

**DOI:** 10.1016/j.bpr.2025.100226

**Published:** 2025-09-05

**Authors:** Samantha S. Romanick, Luis Godoy, Adrian Lopez, Allison Matsumura, Kiana Boc, Travis J. Stewart, Josh E. Baker, Bradley S. Ferguson

**Affiliations:** 1 Cellular and Molecular Pharmacology and Physiology, University of Nevada Reno, Reno, Nevada; 2 Cellular Signal Transduction in the Cardiovascular System COBRE, University of Nevada Reno, Reno, Nevada; 3 Cellular and Molecular Biology, University of Nevada Reno, Reno, Nevada; 4 Department of Nutrition, University of Nevada Reno, Reno, Nevada; 5 Department of Pharmacology, University of Nevada Reno, Reno, Nevada

## Abstract

Skeletal muscle alpha actin (ACTA1) is important for muscle contraction and relaxation, with historical studies focused on ACTA1 mutations in muscle dysfunction. Proteomics reports have consistently observed that actin, including ACTA1, is acetylated at multiple lysine sites. However, few reports have studied the effects of actin acetylation on cellular function, and fewer have examined ACTA1 acetylation on skeletal muscle function. Here, we aimed to examine how ACTA1 acetylation affected actomyosin interactions by determining actin sliding velocity, myosin binding, and calcium sensitivity. In this study, ACTA1 was chemically acetylated via acetic anhydride (AA) to increasing levels of acetylation: low-level acetylation (using 0.1 mM AA), mid-level acetylation (0.3 mM AA), and high-level acetylation (1 mM AA). We report that ACTA1 acetylation significantly decreased actin sliding velocity and actin filament length. Further analysis showed that ACTA1 acetylation significantly increased calcium sensitivity, with a loss of tropomyosin regulation noted with high-level ACTA1 acetylation. Lastly, ACTA1 acetylation enhanced skeletal myosin half maximal binding to actin. These data highlight acetylation as an additional posttranslational modification, outside of phosphorylation, in the regulation of muscle contraction and skeletal muscle alpha actin function.

## Why It Matters

Over the last 50 years, studies have examined how mutations in myofilament proteins affect muscle contraction and relaxation linked to disease. In addition, research focused on how myofilament protein phosphorylation could change muscle function under normal conditions and in response to disease. Recently, acetylation has emerged as a potential regulator for muscle proteins including titin, troponin, and myosin that were shown to worsen cardiac muscle relaxation (titin), improve cardiac relaxation (troponin), or increase muscle contractility (myosin). Recent publications show that skeletal muscle alpha actin is acetylated in cardiac and skeletal muscle. Here, we show that skeletal muscle alpha actin acetylation slows actin sliding velocity, increases actin-myosin binding, and increases calcium sensitivity; these actions would be predicted to change muscle mechanics in vivo.

## Introduction

Acetylation of nonhistone proteins is an emerging topic of interest. Proteomic advancements over the last 15 years show that over 15,000 acetylation sites on more than 4,500 proteins can be modified by acetylation (1,2,3). More recently, research has begun to characterize the functional and physiological importance for protein acetylation outside of nuclear gene regulation (4,5,6,7,8). Indeed, recent studies have begun to focus on mitochondrial and sarcomere protein acetylation in various tissues and organs including the muscle (4,5,6,7,8,9,10).

In muscle, and in particular cardiac muscle, myosin heavy chain, troponin I (cTnI), and titin acetylation have been studied (4,5,6,11,12,13,14). Here, myosin heavy chain acetylation increased contractility, which was postulated to increase cardiac performance in the heart in response to stress (13). More recently, troponin I acetylation was shown to enhance cardiac myocyte relaxation (6,14), whereas conversely titin acetylation was shown to reduce and thus worsen cardiac myocyte relaxation leading to diastolic dysfunction (5). Like titin, actin acetylation appears detrimental to muscle performance. Recent published findings showed that pseudo-acetylation of K326/K328 of the actin gene from *Drosophila melanogaster* (*D. melanogaster*) increased calcium sensitivity (7). In addition, pseudo-acetylation of K326/K328 of the Act57b gene in *D. melanogaster* attenuated flight in flies (8). Human, mouse, and rat skeletal muscle biopsies revealed 12 acetylated lysine residues (K52, K63, K70, K86, K115, K193, K213, K215, K317, K326, K328, and K338) within skeletal muscle alpha actin (ACTA1) (2,15).

ACTA1 has many actin binding proteins and actin interactions. For example, actin-myosin binding is likely to be disrupted by any number of posttranslational modifications (PTMs) such as acetylation. Acetylation of ACTA1 is postulated to result in aberrant contractile function and sarcomere assembly (16). For example, the positively charged amino acid residues K326, K328, and R147 of ACTA1 interact electrostatically with the negatively charged glutamic acid residue E181 of tropomyosin during muscle relaxation. Furthermore, K328 of ACTA1 also interacts electrostatically with E286 of myosin head, subfragment 1 (S1), in the presence of bound rigor myosin (8,17). This would suggest that ACTA1 acetylation would disrupt regulatory switching of tropomyosin and myosin that lead to alterations in contractility.

The thin (actin) and thick (myosin) filaments of the sarcomere are predominantly involved in muscle contraction. The thin filament consists of actin regulated by the troponin complex (inhibitory troponin I, calcium binding troponin C, and tropomyosin binding troponin T) on tropomyosin (18,19,20,21), and the thick filament mainly consists of myosin with several myosin binding proteins (21). In human skeletal muscle, the predominant actin isoform is ACTA1, which encompasses 95% of sarcomeric actin (22). Although historical studies have focused on ACTA1 mutations in ACTA1 function and physiology, no studies have examined ACTA1 acetylation in actin-myosin or actin-tropomyosin interactions.

Thus, in this study, we examined the functional consequence of ACTA1 acetylation on skeletal muscle contractility in vitro. Using in vitro motility assays, we report that ACTA1 acetylation decreased actin sliding velocity and increased calcium sensitivity. In addition, we show that ACTA1 acetylation decreased actin filament length, although this did not affect actin sliding velocity. Lastly, we showed that ACTA1 acetylation increased actin-myosin affinity, but only in the presence of two myosin heads. These findings suggest that ACTA1 acetylation affects actomyosin interactions and highlights acetylation as an additional posttranslational modification outside of phosphorylation in the regulation of muscle contraction.

## Materials and Methods

### Buffers

The following buffers were used: 10× myosin buffer (300 mM KCl, 25 mM imidazole, 1 mM EGTA, 4 mM MgCl_2_, 10 mM dithiothreitol (DTT) (pH to 7.4)); 10× actin buffer (50 mM KCl, 50 mM imidazole, 2 mM EGTA, 8 mM MgCl_2_, 10 mM DTT (pH to 7)); 10× tropomyosin/troponin (TmTn) buffer (1.5 M KCl, 500 mM imidazole, 20 mM EGTA, 80 mM MgCl_2_); and 2× filament buffer (20 mM NaPO_4_ monobasic, 10 mM MgCl_2_, 250 mM NaCl, 2 mM EGTA, 5 mM DTT, 30 nM NaN_3_ (pH to 7)).

Motility buffer for unregulated thin filaments was 1× actin buffer, 0.5% methylcellulose, 1 mM ATP, 10 mM DTT, and oxygen scavenger (292 mg/mL glucose, 1.633 mg/mL glucose oxidase, and 2.25 mg/mL catalase). Motility buffer for regulated thin filaments was 2× filament buffer, add methylcellulose to 0.5%, with 53.8–107.6 μM tropomyosin protein and 70 – 140 μM troponin protein (concentration was doubled in some cases to obtain regulation, further described in the results section). It contains ATP (1 mM) and oxygen scavenger (292 mg/mL glucose, 1.633 mg/mL glucose oxidase, and 2.25 mg/mL catalase). Calcium was added to the concentrations described in the regulated actin motility assays section below.

### Proteins

Rabbit skeletal muscle β myosin (RSM) (23,24) and ACTA1 (25,26,27) were purified from rabbit psoas muscle as described and stored in 50%, by mass, glycerol at −20°C and on ice at 4°C, respectively. Skeletal troponin and tropomyosin (Tm and Tn) were also purified from rabbit psoas muscle as described (28,29). RSM subfragment 1 (S1) proteins were further separated via α-chymotrypsin digest (24,30). Protein concentrations were determined using the following extinction coefficients (0.1% w/v) at 280 nm: 0.53 for RSM (480 kDa) and 0.75 for RSM S1 (130 kDa); 0.55 for BCM (480 kDa) and 0.75 for BCM S1 (130 kDa). 1 μM actin used in in vitro motility (IVM) assays was fluorescently labeled with 0.01 μM tetramethylrhodamine isothiocyanate (TRITC) phalloidin post acetylation via acetic anhydride by overnight incubation (26,27).

### Actin protein modifications

#### Chemical acetylation

Purified ACTA1 protein was prepared as described and polymerized to F-actin before chemical acetylation via acetic anhydride (AA; Sigma Aldrich 320102) incubation (31,32). Three different concentrations of AA were used in this study, 0.1 mM, 0.3 mM, and 1 mM of AA solubilized in methanol. Chemical acetylation was performed as described in Blakeslee et al. (31). AA was prepared and actin acetylation was performed by (1:10) dilution to the final concentrations previously mentioned with 5 μM (for motility assays) and 3 μM (for immunoblot analysis) of purified and polymerized F-actin protein in phosphate buffer solution and incubated at room temperature for 1 hour. Acetylation reactions were quenched by TRITC phalloidin labeling (for motility assays) or the addition of 4× sample buffer (for immunoblot analysis; 200 mM Tris-HCL (pH 6.8), 10% SDS, 40% glycerol, 20% β-mercaptoethanol, and 0.01% bromophenol blue).

#### Enzymatic acetylation

Rabbit ACTA1 was purified as described and polymerized into F-actin. 25 μg of actin was prepared in histone acetyltransferase (HAT) buffer (50 mM tris-HCl, 0.1 mM EDTA, 1 mM DTT (pH to 8)) and incubated with 50 ng of the recombinant HAT enzyme p300/CBP-associated factor (PCAF) per microgram of actin and 25 ng of the recombinant HAT enzyme E1a binding protein P300 (P300) per μg of actin, with or without acetyl-CoA, in a total volume of 100 μL, at 30°C for 1 hour. Reactions were quenched with the addition of 4× sample buffer and boiled at 95°C for 5 minutes before SDS-polyacrylamide gel electrophoresis (PAGE) and immunoblotting (see below) for total acetyl-lysine and ACTA1 (antibody information described in immunoblotting section).

### Immunoblotting

Immunoblot analysis was performed as previously described (33,34). Purified F-actin was acetylated as described above. Proteins were resolved by SDS-PAGE before transfer to nitrocellulose membranes (Bio-Rad). Membranes were blocked using 4% milk and incubated overnight with indicated primary antibodies for skeletal muscle alpha actin rabbit polyclonal antibody (1:1000; Proteintech Group, 17521-1-AP) and rabbit polyclonal acetyl-lysine (1:1000; Cell Signaling Technology, 9441). Membranes were then incubated with horseradish peroxidase (HRP)-conjugated secondary antibodies against the species rabbit (1:2000; Southern Biotech) in milk for 1 hour before chemiluminescence with SuperSignal West Pico (Thermo Scientific) and imaging with ChemiDoc XRS+ imager (Bio-Rad) to detect total protein and acetylation.

### Actin motility assays

#### Flow cell construction

Before performing the in vitro actin motility (IVM) assay, flow cells were prepared. Coverslips (22 mm × 30 mm; Fisherbrand, 12544A) coated with 1.0% nitrocellulose solution (Ladd Research, 10800) were attached to a glass microscope slide (3″ × 1″ × 1 mm; Fisherfinest, 12-544-1) with two layers of ¼-inch double-sided tape (3M), creating a flow channel where solutions can be applied reproducibly.

#### In vitro motility assays

Myosin (100 μg/mL) in 1× myosin buffer was allowed to adhere to the nitrocellulose-coated coverslip by the addition of duplicate washes of 50 μL to the flow cell and incubated at 1 minute each for RSM; the coverslip surface was then blocked with duplicate washes of 50 μL of BSA (5 mg/mL; Sigma, A3059) in actin buffer and incubated at 1 minute each; actin was allowed to bind to myosin by duplicate washes of 50 μL of either TRITC-actin (15 nM) prepared in 1× actin buffer or TRITC-acetylated actin (15 nM) prepared as described in actin buffer and incubated for 1 minute each; this was followed by two washes of actin buffer and two washes of motility buffer for the indicated assay (unregulated and regulated). Flow cells were equilibrated to 30°C before imaging on a Nikon TE2000 epifluorescence microscope (Technical Instruments) and a Roper Cascade 512B camera (Princeton Instruments) with wide field excitation at 575 nm using either 100× or 60× objective. Frames (200–600) and frame rates (1–10 fps) were recorded from three different areas of the flow cell, and the average velocity of a total of 45 moving filaments per flow cell that are larger than 1 μm was measured via manual tracking performed in ImageJ using plugin *mtrackj*. Average velocities and standard deviations were determined using GraphPad Prism. For sliding velocity analysis, *n* >100 filaments/group were analyzed. Experiments were replicated a minimum of three times.

### Cosedimentation assays

Cosedimentation assays were performed via the Cytoskeleton Actin Binding Protein Spin-Down Assay Biochem Kit: Rabbit Skeletal Muscle Actin protocol. Filamentous actin (F-actin) was prepared by manufacturer’s instructions and acetylated as described. RSM and RSM S1 proteins were clarified by centrifugation at 150,000 × *g* for 1 hour at 4°C, and concentrations were prepared as indicated in the results section. Actin (2.5 μM) or acetylated actin (2.5 μM) was incubated with myosin or BSA (2 μM) as a negative control at room temperature for 30 minutes before sedimentation at 14,000 × *g* for 1 hour at 24°C. Supernatants were collected, pellets were resuspended in ultrapure water to a volume of 30 μL, 4× sample buffer was added and boiled at 95°C for 5 minutes, and SDS-Page resolved proteins before Coomassie staining and imaging with ChemiDoc XRS+ imager (Bio-Rad) to detect total protein. Standard curves were constructed from standards prepared using the myosin type indicated in the results section. Concentration of proteins present in each band were determined using a standard curve, and Bmax (maximum specific binding), Kd (half maximum binding), and h (Hill slope) results were obtained by best fit to the Hill equation using GraphPad Prism.

### Actin-activated ATPase assays

As described by Chifflet et al. (35), actin-activated ATPase activity was measured at 30°C. 25 μM actin (with rabbit skeletal myosin; RSM) was prepared in assay buffer (10 mM MOPS (pH 7), 50 mM NaCl, 1 mM DTT, 5 mM MgCl_2_, 0.1 mM EGTA, and 30 nM NaN_3_) with 2 mM ATP, and 0.3 mg/mL rabbit skeletal myosin was prepared in a no-salt assay buffer (10 mM MOPS (pH 7), 1 mM DTT, 5 mM MgCl_2_, 0.1 mM EGTA, and 30 nM NaN_3_). Reactions were initiated with the addition of myosin with a final actin concentration of 20 μM with RSM (0.06 mg/mL). Reactions were quenched with the addition of 5% SDS, and methanol (1:12.5) was added to remove bubbles before determining phosphate. Phosphate was determined as previously described (36).

### Regulated actin motility assays

In vitro actin motility assays were performed as described in the text with the substitution of regulated actin thin filaments (RTFs) for F-actin and the addition of calcium. For reconstitution of RTFs, 15 nM TRITC-phalloidin-labeled F-actin was incubated with 0.25–0.5 nM troponin and tropomyosin (concentration was doubled in some cases to obtain regulation, further described in the results section) on ice in 1× TmTn buffer for 20+ minutes. Incubation times were longer for the acetylated actin (0.3 mM and 1 mM) compared with the shorter incubation time with less acetylated actin (ACTA1 and 0.1 mM acetylated ACTA1). Longer incubation times were necessary to obtain regulation of the thin filament. Regulation was determined by the lack of motility at pCa 10 (no calcium added). Calcium and RTF were used in the in vitro motility assays to measure RTF velocity and determine calcium sensitivity. Calcium concentration at pCa (log_10_[Ca^2+^]) 4, 5, 6, 7, and 10 was determined using the free calcium calculator (Ca-EGTA Calculator, UC Davis), where pCa 4 indicates maximum calcium concentration (positive control), and pCa 10 indicates no calcium (negative control).

### Statistical analysis

Statistical analysis was performed by Tukey’s post hoc analysis, one-way ANOVA for deacetylated versus acetylated ACTA1 results, unless otherwise stated in the materials and methods or results sections, using GraphPad Prism (GraphPad Software). *p*-values <0.05 indicated statistical significance.

## Results

### Skeletal muscle alpha actin acetylation attenuates actin sliding velocity and actin filament length

**Figure 1:**
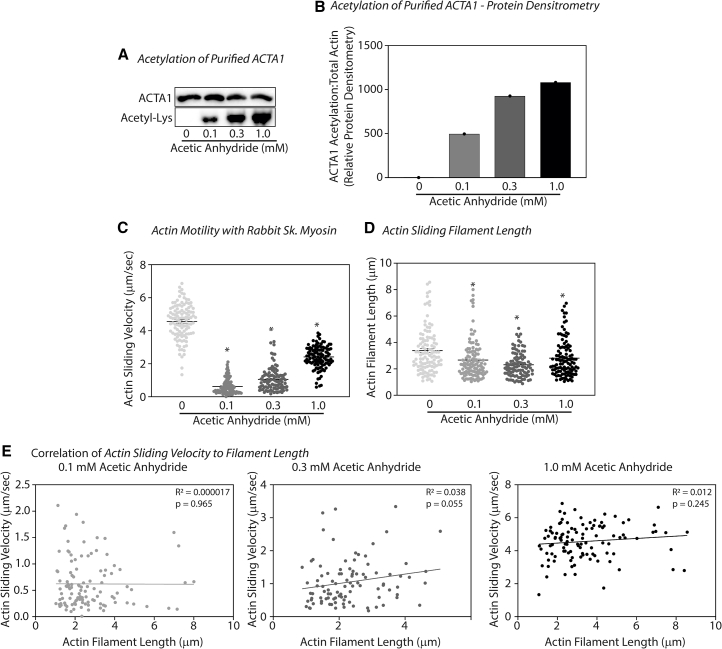
ACTA1 acetylation inhibits actin sliding velocity and reduces actin filament length. (*A*) Purified ACTA1 protein was incubated with increasing concentrations of acetic anhydride, before immunoblot analysis for total ACTA1 and acetyl-lysine. (*B*) In vitro motility assays were performed as described to assess sliding velocity of nonacetylated ACTA1 (*light gray*) or ACTA1 acetylated with 0.1 mM (*gray*), 0.3 mM (*dark gray*), and 1 mM (*black*) acetic anhydride. (*C*) Actin filament length was assessed post motility with RSM. (*D*) The correlation between sliding velocity and filament length was measured using linear regression with the best fit determined by the R^2^ value with *n* = 33–109 filaments analyzed for each group: 0.1 mM (*left*), 0.3 mM (*middle*), and 1 mM (*right*) acetic anhydride. For sliding velocity analysis, *n* > 100 filaments/group were analyzed. Experiments were replicated a minimum of three times. Statistical significance was determined via one-way ANOVA with Tukey’s post hoc for sliding velocity and filament length (*p* < 0.05) or Pearson’s correlation (*p* < 0.05).

Published proteomics showed that ACTA1 can be acetylated on many different lysine residues (15,33). Yet how ACTA1 acetylation affects actin-myosin and actin-tropomyosin binding and actin sliding velocity remains less clear (7,8). As such, we chemically acetylated purified ACTA1 using AA and examined deacetylated versus dose-dependent increases in ACTA1 acetylation on actin sliding velocity and filament length using in vitro motility assays. AA acetylates proteins on primary amines (37,38,39), such as lysine residues. Here, we incubated purified ACTA1 with increasing doses of 0.1 mM, 0.3 mM, and 1 mM AA to achieve low, mid, and high levels of acetylation, respectively (Fig. 1 *A*). AA did not change total ACTA1 protein levels (Fig. 1 *A*).

We also showed that recombinant lysine acetyltransferase (KAT) enzymes P300 and PCAF acetylate ACTA1 in vitro (Fig. S1). KATs acetylate protein targets by transferring an acetyl group from acetyl-CoA onto the epsilon amino group of lysine residues (40). Published reports show that PCAF can localize to the sarcomere of muscle (11). Combined, these findings suggest functional importance for acetyl transferases in ACTA1 acetylation.

We next examined actin sliding velocity under nonregulated conditions (i.e., only actin and myosin were present). Rabbit skeletal muscle consists of ACTA1, which is highly conserved among species. In human skeletal muscle the predominant myosin isoform is the fast myosin IIa isoform, which is also predominantly found in rabbit skeletal muscle (41,42). Here, we report that rabbit ACTA1 acetylation decreased actin sliding velocity (Fig. 1 *B*). Interestingly, we noted that low-dose AA significantly attenuated actin sliding velocity compared with nonacetylated ACTA1 and that higher doses of AA, whereas inhibitory, had less of an effect on sliding velocity (Fig. 1 *B*). These data suggest that hyperacetylation with 1 mM AA affects actin-myosin interactions in a manner that mitigates changes to sliding velocity.

Velocity of the unidirectional movement of F-actin is determined by distance traveled in microns using frame rate over time in seconds (μm/sec). The velocity of ACTA1 moving across RSM was 4.562 ± 1.062 μm/sec. However, when ACTA1 was acetylated, a significant decrease in velocity was observed, with low-level ACTA1 acetylation velocity of 0.630 ± 0.455 μm/sec, mid-level ACTA1 acetylation velocity of 1.050 ± 0.683 μm/sec, and high-level ACTA1 acetylation velocity of 2.411 ± 0.669 μm/sec with RSM.

In addition to changes in actin sliding velocity, we further report that ACTA1 acetylation decreased actin filament length (Fig. 1 *C*). However, unlike sliding velocity, there were no dose-dependent, or acetyl-dependent, changes observed for actin filament length. In short, low levels and high levels of ACTA1 acetylation led to similar decreases in filament length. Here, filament length was determined post motility. As a next step, we used linear regression of actin sliding velocity and filament length to determine if sliding velocity decreased due to decreased filament length. Previous data suggest that we would not expect any change in actin sliding velocity with shorter actin filament lengths (longer than 1 μm) if there was a decrease in adhered myosin density (43,44). Consistent with these findings, actin filament length did not correlate to changes in actin sliding velocity at any level of acetylation (Fig. 1 *D*). For all AA concentrations, the R^2^ value of the linear relationship between actin sliding velocity and actin filament length was close to zero (Fig. 1 *D*). Combined, these data suggest that ACTA1 acetylation slows actin sliding velocity through a mechanism independent of changes to actin filament length.

### ACTA1 acetylation affects actomyosin binding in cosedimentation assays when two myosin heads are present

The decrease in sliding velocity with ACTA1 acetylation suggests a few possibilities: 1) acetylated ACTA1 is bound so strongly to myosin that the binding of ATP to myosin is not able to dissociate the complex as rapidly; 2) ADP release is slowed; or 3) acetylation of ACTA1 affects its ability to bind strongly to myosin. To address these, we examined the binding of ACTA1 and acetylated ACTA1 to RSM using in vitro cosedimentation assays, as well as tested actin-activated ATPase activity to determine if acetylation of ACTA1 affects the ATP hydrolysis rate.

**Figure 2:**
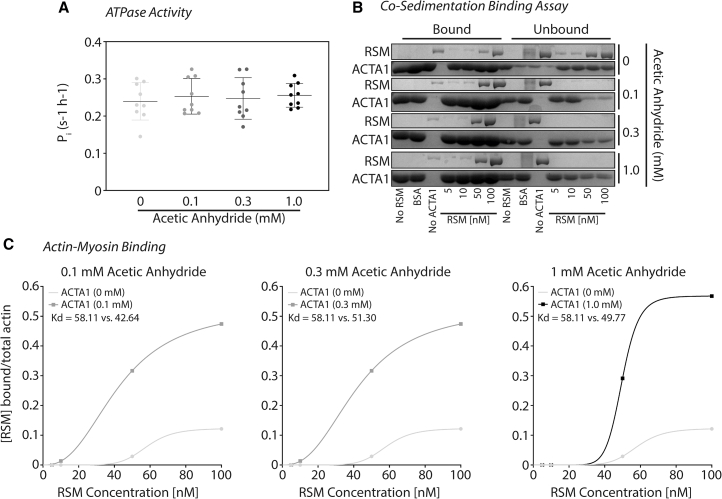
ACTA1 acetylation does not change ATPase activity but increases actin-myosin binding in cosedimentation assays with intact skeletal myosin. (*A*) In vitro actin-activated ATPase assays were performed as described, and the release of P_i_ was measured per second per head (s^−1^ h^−1^) of RSM with nonacetylated ACTA1 (*light gray*), ACTA1 acetylated with 0.1 mM (*gray*), 0.3 mM (*dark gray*), or 1 mM (*black*) acetic anhydride; an *n* = 9 for each group was assessed. One-way ANOVA with Tukey’s post hoc analysis was used to determine significance (*p* < 0.05). (*B*) Cosedimentation assays were performed as described to determine RSM binding to nonacetylated ACTA1 (0 mM acetic anhydride) or ACTA1 acetylated with 0.1 mM, 0.3 mM, or 1 mM acetic anhydride. (*C*) Densitometry was performed to examine bound RSM to determine Kd.

First, we used an actin-activated steady-state ATPase activity assay (31) to determine the maximum rate of inorganic phosphate (P_i_) release (36) from RSM with ACTA1 and various levels of acetylated ACTA1. We report no changes in the ATPase activity of RSM between nonacetylated ACTA1 with any level of ACTA1 acetylation in response to increasing concentrations of AA (Fig. 2 *A*).

Next, we performed cosedimentation assays to examine actomyosin interactions. Myosin and myosin subfragment 1 (S1) binding to ACTA1 or acetylated ACTA1 was quantified and determined by the dissociation constant (Kd) (45,46). Specific binding was determined by total bound myosin or S1 (found in the sediment) normalized to total ACTA1 concentration in the assay. ACTA1 and increasing levels of acetylated ACTA1 were allowed to bind to RSM or S1. Myosin subfragment 1 is the globular domain of myosin where actin binding takes place as well as ATP binding and hydrolysis.

The rate of actin-myosin binding and the free energy of actin-myosin binding increase with ionic interactions (7,8). From this, we would expect to see stronger binding of skeletal myosin to acetylated actin, as acetylation neutralizes lysine’s positive charge on actin; this causes less repulsion because the actin-binding site on myosin is positively charged. Indeed, we show an increase in skeletal myosin binding to low-level acetylated ACTA1 (Kd = 42.64 nM), mid-level acetylated ACTA1 (Kd = 51.30 nM), and high-level acetylated ACTA1 (Kd = 49.77 nM) compared with nonacetylated ACTA1 (Kd = 58.11 nM) (Fig. 2, *B* and *C*).

**Figure 3:**
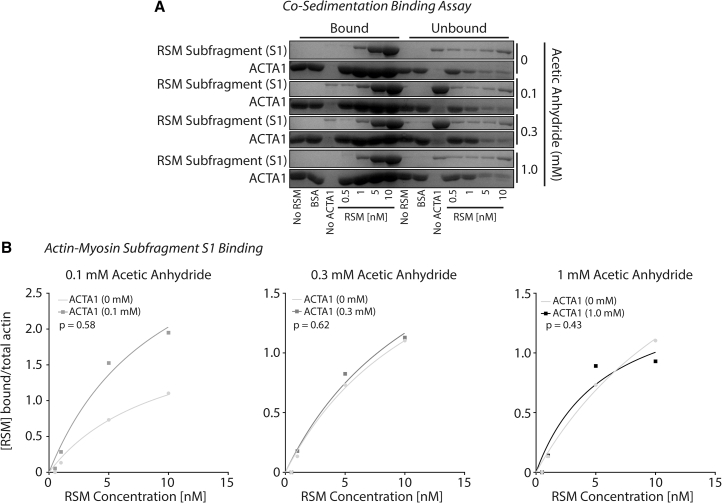
ACTA1 acetylation does not increase myosin binding with subfragment S1. (*A*) Cosedimentation assays were performed as described to determine RSM subfragment 1 (sS1) binding to nonacetylated ACTA1 or ACTA1 acetylated with 0.1 mM, 0.3 mM, or 1 mM acetic anhydride. (*B*) Densitometry was performed, and bound RSM sS1 was fit to the Hill equation to determine Kd. No significant changes were noted in actin-myosin binding (*p* > 0.05).

Loop 2 of skeletal S1 contains a “K-site,” so called for its lysine-rich region that lies between amino acid residues 633 and 642 (G G K_1_ K_2_ G G K_3_ K_4_ K_5_ G) (47,48). This sequence appears important for actin binding to skeletal myosin. As our data above suggest that ACTA1 acetylation increases binding affinity to skeletal myosin, we would postulate similar findings with the K-site on skeletal S1. Although Kd was slightly less for acetylated ACTA1 compared with nonacetylated ACTA1, contradictory to our postulate, we observed no significant differences (*p* > 0.4) (Fig. 3, *A* and *B*). Altogether, these data suggest that acetylated ACTA1 increases binding affinity to intact RSM but not S1.

### ACTA1 acetylation increases calcium sensitivity

Thus far, we have shown that acetylation of ACTA1 affects actomyosin binding with unregulated ACTA1; therefore, our next aim was to examine the actomyosin interaction with troponin- and tropomyosin-regulated ACTA1 and acetylated ACTA1 using the in vitro motility assay. For this, we used troponin-tropomyosin-regulated nonacetylated ACTA1 and acetylated ACTA1 (using increasing AA concentrations) in the in vitro motility assay to determine actin sliding velocity against the log scale of calcium concentrations, where log_10_4 (pCa 4) is maximum calcium concentration, and log_10_10 (pCa 10) has no free calcium present. We expect maximum velocities at pCa 4 and no velocity at pCa 10.

**Figure 4:**
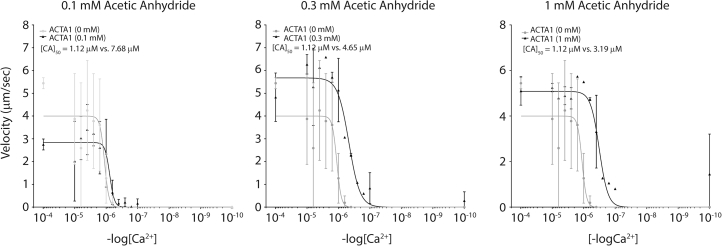
ACTA1 acetylation increased calcium sensitivity under troponin/tropomyosin regulation. In vitro motility assays were used to determine calcium sensitivity of nonacetylated ACTA1 (0 mM), or ACTA1 acetylated with 0.1 mM (*left*), 0.3 mM (*middle*), or 1mM (*right*) of acetic anhydride with RSM in the presence of troponins and tropomyosin with increasing calcium concentrations. pCA calculations are noted for nonacetylated ACTA1 (1.12 μM), ACTA1 acetylated with 0.1 mM (7.68 μM), 0.3 mM (4.65 μM), or 1 mM (3.19 μM, respectively) acetic anhydride; an *n*> 90 filaments was analyzed for each group.

We examined regulated nonacetylated ACTA1 thin filament velocity on a scale of calcium concentrations with RSM and determined the calcium sensitivity (pCa50 = 1.12 μM) (Fig. 4). We then determined calcium sensitivity for regulated low-level acetylated ACTA1 (pCa50 = 7.68 μM), mid-level acetylated ACTA1 (4.65 μM), and high-level acetylated ACTA1 (pCa50 = 3.19 μM) with RSM (Fig. 4). Interestingly, we noted striking differences between low-level, mid-level, and high-level ACTA1 acetylation with regard to regulated sliding velocity. Low-level acetylated ACTA1 had decreased actin sliding velocity, which mirrored unregulated findings in Fig. 1. However, mid-level and high-level acetylated ACTA1 had increased actin sliding velocity. Published reports show that pseudo-acetylated Act57B (*D. melanogaster* muscle actin) increased calcium sensitivity with no change in actin sliding velocity (7). From this, we conclude that ACTA1 acetylation increased calcium sensitivity, with differences noted in actin sliding velocity dependent on the acetylation status of ACTA1. Further, these data suggest that increased ACTA1 acetylation interferes with tropomyosin binding.

## Discussion

**Figure 5:**
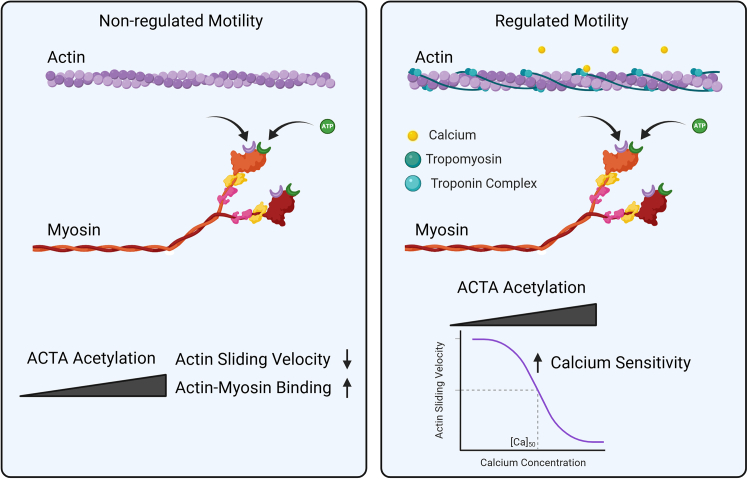
Model showing that ACTA1 acetylation changed actin-myosin interactions and increased calcium sensitivity under nonregulated and regulated conditions. Nonregulated condition infers that actin-myosin and ATP were analyzed without other myofilaments. Regulated condition includes examination of actin sliding velocity with actin, troponins, tropomyosin, myosin, and ATP with increasing concentrations of calcium (Ca^2+^). Created in https://BioRender.com.

In this study, we showed that ACTA1 acetylation enhanced myosin binding and increased calcium sensitivity. Here, ACTA1 acetylation affects actomyosin dynamics by reducing in vitro motility and increasing skeletal myosin binding under nonregulated conditions, whereas it increased in vitro calcium sensitivity*,* with a loss of tropomyosin binding under regulated conditions (Fig. 5). Multiple published proteomics reports demonstrate that ACTA1 can be acetylated (2,33), and thus, these data suggest that acetylation serves as a regulatory PTM for skeletal muscle contraction. However, further investigation for the functional role of ACTA1 acetylation in skeletal muscle contraction in animal models is still needed.

Top-down mass spectrometry has identified numerous protein modifications on sarcomere proteins that include deamidation, methylation, tri-methylation, S-gluthaionylation, and acetylation (49,50). Thus, sarcomeric posttranslational modifications likely play an important role in the regulation of contractile muscle function and/or dysfunction, yet our understanding of these modifications in muscle regulation remains fragmentary. Published reports demonstrate that myosin heavy chain acetylation increases contractility (13), and troponin I acetylation enhances cardiac myocyte relaxation (6,14), whereas titin acetylation reduces cardiac myocyte relaxation that contributes to diastolic dysfunction (5). In addition, published findings show that pseudo-acetylation of K326/K328 of the actin gene from *D. melanogaster* increased calcium sensitivity (7), and pseudo-acetylation of K326/K328 of the Act57b gene in *D. melanogaster* attenuated flight in flies (8). We published that ACTA1 could be acetylated at K52, K317, and K328 in the cardiac muscle of obese mice (33). Combined, these reports support the postulate that sarcomere protein acetylation is an important modification for muscle regulation. Consistent with this postulate, our in vitro data demonstrate that ACTA1 acetylation affects actomyosin motility and tunes calcium sensitivity, suggesting an important role for ACTA1 in skeletal muscle function and that PTMs of ACTA1 can have profound physiological consequences.

Viswanathan et al. (8) previously suggested that K326 and K328 of the actin gene interact with both myosin and tropomyosin electrostatically. Moreover, Barua et al. (51) identified tropomyosin binding sites on actin that are evolutionarily conserved, D25, E334, K326, and K328. For these studies, Barua et al. mutated tropomyosin binding sites on actin to alanine, which prevented actin binding to tropomyosin and reduced in vitro motility (51). These findings suggest that these actin residues are important for tropomyosin and myosin binding and support the postulate that PTMs of actin can greatly affect actin-tropomyosin interactions. Indeed, we showed that chemical acetylation of ACTA1 yielded consistent results with Barua et al., in which ACTA1 acetylation reduced in vitro motility under nonregulated conditions and resulted in increased calcium sensitivity likely due to decreased tropomyosin binding. Interestingly, as ACTA1 acetylation increased, decreases to actin sliding velocity were attenuated (Fig. 1 *C*), suggesting that higher levels of ACTA1 acetylation by AA have a different role with actin-myosin interactions compared with lower levels of ACTA1 acetylation. Consistent with this, low levels of ACTA1 acetylation by AA (0.1 mM) led to decreased sliding velocity under regulated conditions when troponins and tropomyosin were present, whereas high levels of ACTA1 acetylation (>0.3 mM AA) increased actin sliding velocity (Fig. 4). Although these contrasting differences are interesting, it is important to note that we observed a normalization (i.e., coming back to baseline) of actin sliding velocity under nonregulated conditions, which may suggest that the inclusion of troponins and skeletal tropomyosin changes the actin-myosin dynamics at higher acetylation states to favor increased sliding velocity.

Schmidt et al. (7) showed that chemically acetylated actin did not decrease actin sliding velocity under nonregulated or regulated conditions. In addition, ACTA1 acetylation did not inhibit actin-tropomyosin binding, yet actin acetylation increased calcium sensitivity, and there was an increase in the percent of motile filaments with acetylation suggesting that tropomyosin inhibition of myosin binding to actin was decreased (7). It is interesting to postulate differences between Schmidt et al. on actomyosin sliding velocity with our findings. One could speculate that differences observed might stem from the lower stoichiometric molar ratios used by Schmidt et al. for chemical actin acetylation (1:1 and 80:1 mole AA to actin) compared with our higher stoichiometric molar ratio (60:1 and 200:1). Densitometry from Schmidt approximated a 300-fold increase in acetylation (7). Although densitometry can differ depending on the exposure settings, we observed an approximately 1,000-fold increase with our highest doses of AA, demonstrating higher levels of acetylation. Secondly, Schmidt et al. (7) examined actin acetylation sites with proteomics and observed 12 distinct lysine acetylation sites with 80:1 molar ratio of AA to actin, yet AA can acetylate some arginine residues (37,38,39,52). Thus, it is plausible that hyperacetylation of actin with AA drives lysine and arginine acetylation, affecting actomyosin and actin-tropomyosin interactions. Lastly, it is important to note that Schmidt et al. (7) used cardiac tropomyosin, whereas we used skeletal tropomyosin isolated from rabbit psoas muscle in our actin motility assays. As the rabbit psoas muscle consists heavily of fast twitch muscle fibers compared with cardiac tissue (53), it is possible that this contributes to the differences observed in actin-tropomyosin and actin-myosin binding and sliding velocity. Although our groups differ in relation to actomyosin sliding velocity, we observe similarities with increased calcium sensitivity. Future studies will need to tease apart a role for individual lysine residue acetylation in actomyosin and actin-tropomyosin interactions as well as differences between cardiac and skeletal troponins and tropomyosin.

As mentioned, we identified ACTA1 to be acetylated at K52, K317, and K328 (33). Although K317 and K328 sit in close proximity to glutamic acid (E) residues E286 on myosin and E181 of tropomyosin (8), K52 sits in close proximity to aspartic acid (D) 391 within myosin binding protein-C (MyBP-C) (54). MyBP-C is critical for myosin-actin interactions and tropomyosin dissociation from myosin binding sites on actin (54,55). Loss of MyBP-C could serve to weaken the muscle, with mutations in cardiac MyBP-C linked to dilated cardiomyopathy and contractile dysfunction (56,57). Reports also suggest that cytoskeletal actin acetylation at K50 can inhibit actin polymerization mediated by inverted formin 2 (47,58). Although this could suggest that acetylation inhibits polymerization to decrease actin filament length, enzymatic polymerization/depolymerization is not at play in our ex vivo setting. Another postulate could thus be that ACTA1 acetylation increases actin-filament break rate as actin interacts with myosin in our motility assays. This could suggest that ACTA1 acetylation destabilizes filamentous actin, although actin break rate was not examined in our studies. It is important to note that like K326 and K328, K52 of ACTA1 is consistently observed in proteomic datasets (2), and it is evolutionarily conserved (59). Collectively, this would suggest that K326 and K328 acetylation could affect ACTA1 contractility, whereas K52 of ACTA1 may disrupt tropomyosin dissociation from actin, due to changes in MyBP, as well as actin contributing to decreased actin filament length and perhaps increased actin break rate, although these hypotheses still need testing.

### Considerations and limitations

It is important to note that much of this work involved the isolation of purified ACTA1. These isolation methods have the potential to minimize basal acetylation levels. Basal levels of many PTMs occur physiologically, as indicated from proteomics reports (15,48,60), and basal expression of protein acetylation is no different, as basal levels of tubulin acetylation, for example, have been noted in the myocardium of standard chow-fed mice (61,62). Many PTMs, including acetylation, are dynamic and reversible through the actions of “eraser” and “writer” proteins; for acetylation, this includes histone deacetylases and HATs (63). Changes in this dynamic and reversible action can contribute to cardiac dysfunction, where, for example, hyperacetylation of titin was shown to promote cardiac stiffness and worsen cardiac relaxation in a rodent model of diastolic heart failure (5). Keeping with this, Fig. 1 gives the impression that basal acetylation of ACTA1 is zero after our isolation techniques. However, overexposure of Fig. 1 demonstrates that there is basal ACTA1 acetylation despite isolation techniques (Fig. S2). In this case, the perceived limitation would be the increased hyperacetylation with AA, which at the highest dose does not likely represent true physiology.

Given the above, we should further note an important limitation within our study design, which is the hyperacetylation of lysine residues that would likely not occur in an intact native protein in vivo. Indeed, mouse ACTA1 has 19 lysine residues, yet many proteomics reports highlight acetylation of actin on K52, 63, 70, 86, 115, 317, 328, and 330 (15,64), suggesting that actin acetylation at all 19 residues does not occur, likely due to inaccessibility of lysine to writer proteins (i.e., HATs) to those sites. Here, the addition of AA to our purified ACTA1 could potentially target more lysine residues than under physiological conditions. However, it should be noted that for actin motility assays, we are dealing with intact filamentous actin in its more native state, and thus, although AA is likely targeting more actin molecules for acetylation than under normal physiological conditions (stoichiometry differences), we are not necessarily targeting inaccessible lysine residues. Still, the changes in stoichiometry observed in vivo versus our ex vivo experimental design would likely lead to a more robust phenotype that may not be as clearly observed in vivo. Lastly, it is important to note that lysine acetylation can also be impacted through protein-protein interactions observed in vivo. For example, actin bound to troponins and tropomyosin may block lysine residues from HATs and histone deacetylases that would affect acetylation. As such, acetylation of purified ACTA1 ex vivo may lead to acetylation of sites typically inaccessible under physiological conditions.

Given these limitations, our data still show that acetylation of ACTA1 can have functional consequences to actin sliding velocity, actin-myosin binding, and calcium sensitivity. Moreover, proteomics reports show that actin is acetylated in vivo. Together, these studies highlight the need for physiological examination of ACTA1 acetylation in vivo under normal and diseased conditions; this can be achieved with traditional site-directed mutagenesis to derive acetyl-mimic and acetyl-dead ACTA1 animal models (8) or with newer tools, such as genetic code expansion to add acetyl-groups to proteins in vivo, which has primarily been used in bacteria (65) and more recently *Drosophila* (66).

## Acknowledgments

This work is supported by the Dennis Meiss & Janet Ralston Fund for Nutri-epigenetic Research, the 10.13039/100000057National Institute of General Medical Sciences (NIGMS) of the NIH (P20 GM130459), the 10.13039/100000050National Heart, Lung, and Blood Institute of the NIH (R15 HL143496), and the 10.13039/100000049National Institute on Aging of the NIH (R21 AG077248) to B.S.F. Core facilities used for research were supported by 10.13039/100000057NIGMS of the NIH (P20 GM130459).

## Author contributions

S.S.R., L.G., A.L., A.M., K.B., and T.J.S. performed research. S.S.R., L.G., A.M., K.B., T.J.S., J.E.B., and B.S.F. analyzed data. S.S.R., J.E.B., and B.S.F. designed the research experiment. S.S.R., L.G., T.J.S., J.E.B., and B.S.F. wrote the paper.

## Declaration of interests

The authors declare no conflicts of interest.

## Supporting Materials

Document S1. Figures S1 and S2

Document S2. Article plus Supporting Material
