# Supplementary material for: Skeletal muscle alpha actin acetylation enhances myosin binding and increases calcium sensitivity
Source: Biophys Rep (N Y). 2025 Sep 5;5(4):100226. doi: 10.1016/j.bpr.2025.100226 (PMC12478086; doi:10.1016/j.bpr.2025.100226)
Supplement: Document S1. Figures S1 and S2 [file mmc1.pdf]

Biophysical Reports, Volume *5*

Supplemental information

Skeletal muscle alpha actin acetylation enhances myosin binding and increases calcium sensitivity

Samantha S. Romanick, Luis Godoy, Adrian Lopez, Allison Matsumura, Kiana Boc, Travis J. Stewart, Josh E. Baker, and Bradley S. Ferguson

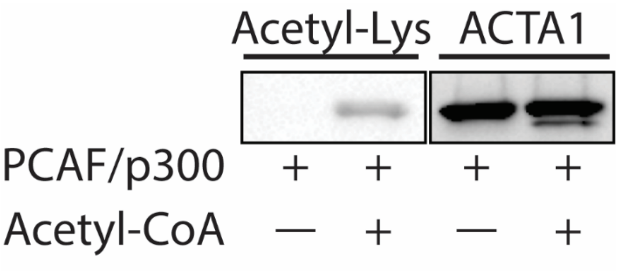

Supplemental Figure 1. PCAF/p300 acetylate purified ACTA1. Purfied ACTA1 protein was incubated in the absence or presence of 50ng of recombinant histone acetyltransferase enzyme p300/CBP-associated factor (PCAF) per µg of actin and 25ng of the recombinant histone acetyltransferase enzyme E1a binding protein P300 (P300) per µg of actin, with or without Acetyl-CoA at 30ºC for 1 hour prior to immunoblotting acetyl-lysine and total ACTA1.

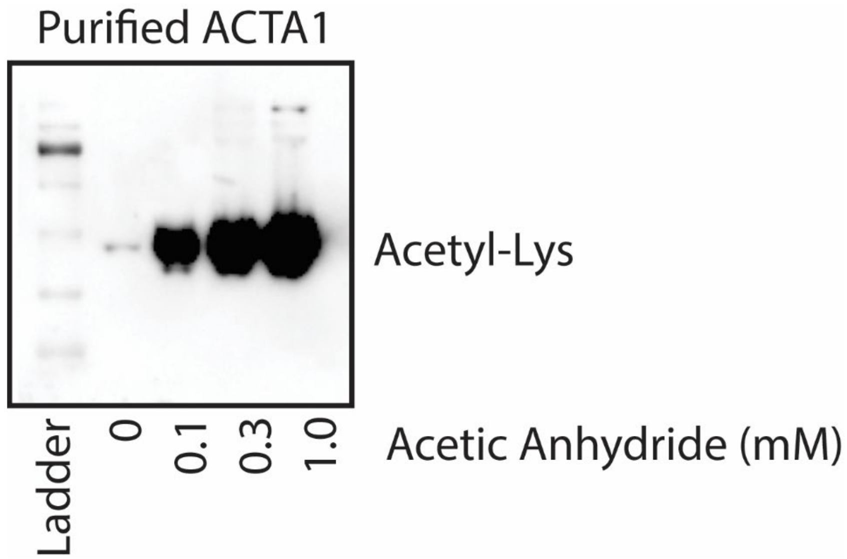

Supplemental Figure 2. Purified ACTA1 is acetylated with acetic anhydride increasing ACTA1 acetylation. Purified ACTA1 protein was incubated with increasing concentrations of acetic anhydride, prior to immunoblot analysis for acetyl-lysine. The whole image is shown.
